# Ozone therapy for high-grade glioma: an overview

**DOI:** 10.3389/fonc.2023.1161206

**Published:** 2023-05-24

**Authors:** Li Yanchu, Pu Rong, Cao Rong, Zhang Li, Yang Xiaoyan, Wang Feng

**Affiliations:** ^1^ Head and Neck Oncology Ward, West China Hospital of Sichuan University, Chengdu, China; ^2^ Oncology Department, Chengdu Fuxing Hospital, Chengdu, China; ^3^ Radiation Therapy Department, West China Hospital of Sichuan University, Chengdu, China

**Keywords:** ozone, high-grade glioma, therapy, chemotherapy, radiation therapy

## Abstract

High-grade gliomas (grades III and IV) are highly malignant and aggressive brain tumors that present significant treatment challenges. Despite advances in surgery, chemotherapy, and radiation therapy, the prognosis for patients with glioma remains poor, with a median overall survival (mOS) range of 9–12 months. Therefore, exploring new and effective therapeutic strategies to improve glioma prognosis is of utmost importance and ozone therapy is a viable option. Ozone therapy has been used in various cancers, such as colon, breast, and lung, yielding significant results in preclinical studies and clinical trials. Only a few studies have been conducted on gliomas. Furthermore, since the metabolism of brain cells involves aerobic glycolysis, ozone therapy may improve the oxygen condition and enhance glioma radiation treatment. However, understanding the correct ozone dosage and optimal time of administration remains challenging. Herein, we hypothesize that ozone therapy should be more effective in gliomas compared with other tumors. This study provides an overview of the use of ozone therapy in high-grade glioma, including mechanisms of action, preclinical data, and clinical evidence.

## Introduction

High-grade glioma is a type of malignant brain tumor typically characterized by aggressiveness, resistance to classic treatments, and poor prognosis. Despite advances in surgery, chemotherapy, and radiation therapy (RT), the survival rate among patients with glioma remains low, with a median overall survival (mOS) range of 9–12 months. Therefore, it is important to explore alternative and complementary treatments, including ozone therapy, for gliomas.

Ozone therapy has been discussed and used in medical care for almost 60 years (1). The first report on ozone therapy, which was used as ozonated autohemotherapy (O_3_-AHT), was published by Wolff in 1974 (2). The therapy, also known as oxidative therapy, involves the administration of ozone, a form of oxygen, into the body. The therapeutic efficacy of ozone may partly rely on controlled and moderate levels of oxidative stress produced by ozone reactions, and the critical balance between the effectiveness and toxicity of ozone may be dependent on the degree of oxidative stress (3). Additionally, ozone can increase the oxygen concentration in tissues, boost the immune system, and induce antimicrobial effects capable of producing chromosome breakages in human cell cultures (4). As such, ozone therapy has been used to treat a variety of medical conditions, including cancer (5–8).

From the literature, ozone therapy has been used as medical treatment with the purpose of inducing apoptosis, reducing tumor cell proliferation, inhibiting migration and invasion, reducing angiogenesis, and enhancing the efficacy of conventional chemotherapy and radiation therapy in different types of cancer (9). Although the effects of ozone in several types of cancers have been reported, only a few studies were conducted on gliomas. In this review, we summarize and discuss the use of ozone in high-grade gliomas, including its mechanisms of action, preclinical data, and clinical evidence.

## Literature search method

We searched Medline (*via* PubMed) and Google Scholar for preclinical and clinical studies utilizing ozone treatment for gliomas. No limitations regarding publication time, study type, or sample units were applied to the search, with all relevant studies included. The search was conducted using a combined filter and the following medical subject headings (MeSH) terms: [‘Ozone/glioma’ (Mesh) OR ‘Ozone/glioblastoma’ (Mesh) OR ‘Ozone/astrocyte’ (MeSH) AND ‘Ozone/oligodendrocyte’ (MeSH) AND ‘Ozone/cancer’]. We screened titles and abstracts independently and evaluated full texts of all relevant articles.

## Mechanisms of ozone therapy in cancer

The potential mechanisms of ozone therapy were reviewed in this study. Ozone is a highly reactive gas, and its principal therapeutic effects come from its ability to increase oxygen levels and induce oxidative stress in affected tissues (10–12). According to previous preclinical studies, ozone can inhibit cancer growth and is a sufficient anticancer treatment; however, its mechanisms are not fully understood and more research is required to determine its exact pathways. Thus, it is not widely used or approved for clinical cancer treatment.

According to previous studies, although tumor cells require a large supply of oxygen for growth and proliferation, the abnormal structure and function of tumor blood vessels often leads to hypoxia within the tumor microenvironment. Meanwhile, hypoxia further increases oxidative stress by activating the hypoxia-inducible factor (HIF) pathway, which promotes the expression of genes involved in angiogenesis, cell survival, and metabolism. In brain tissue, chronic inflammation can induce oxidative stress in astrocytes and microglia. Simultaneously, reactive oxygen species (ROS) can promote cancer initiation and progression, particularly in glioblastoma (GBM) treatment, including with *Temozolomide* (TMZ), eliciting oxidative stress, which modifies the status of DNA methylation in neoplastic cells (13, 14). Moreover, GBM has a high metabolic rate, which results in increased basal levels of ROS and the generation of an immunosuppressive tumor environment. Thus, oxidative stress and ROS levels are associated with glioma development (15, 16).

However, ozone could generate high ROS level and oxygen, which could induce an ROS anticancer effect and relieve hypoxia (3, 17). Ozone-dependent ROS-mediated fatty acid oxidation is involved in the formation of lipid ozonation products (LOPs), which act as signal transducers by triggering ROS signaling and have been recognized as key factors in monitoring the positive or negative effects of oxidative compounds. These processes are associated with Nrf2/Keap1/ARE and AMPK/FOXO/mTOR/Sir1 pathways and Nrf2/NF-κB crosstalk, which are ultimately activated *via* moderate oxidative stress and act on NF-κB expression and inflammatory responses (3). Moreover, ROS and LOPs are associated with multiple oxidative/reduction biochemical reactions that occur in human cells, such as the HIF-1α pathway, HO-1 signaling, and NO/iNOS biochemical machinery (1, 18, 19). Hence, ozone therapy can improve blood circulation and oxygenation of neoplastic tissues, ameliorate metabolism, improve chronic oxidative stress, activate the immune and neuroendocrine systems, inhibit growth and metastasis, and induce tumor necrosis (20–23).

**Figure 1:**
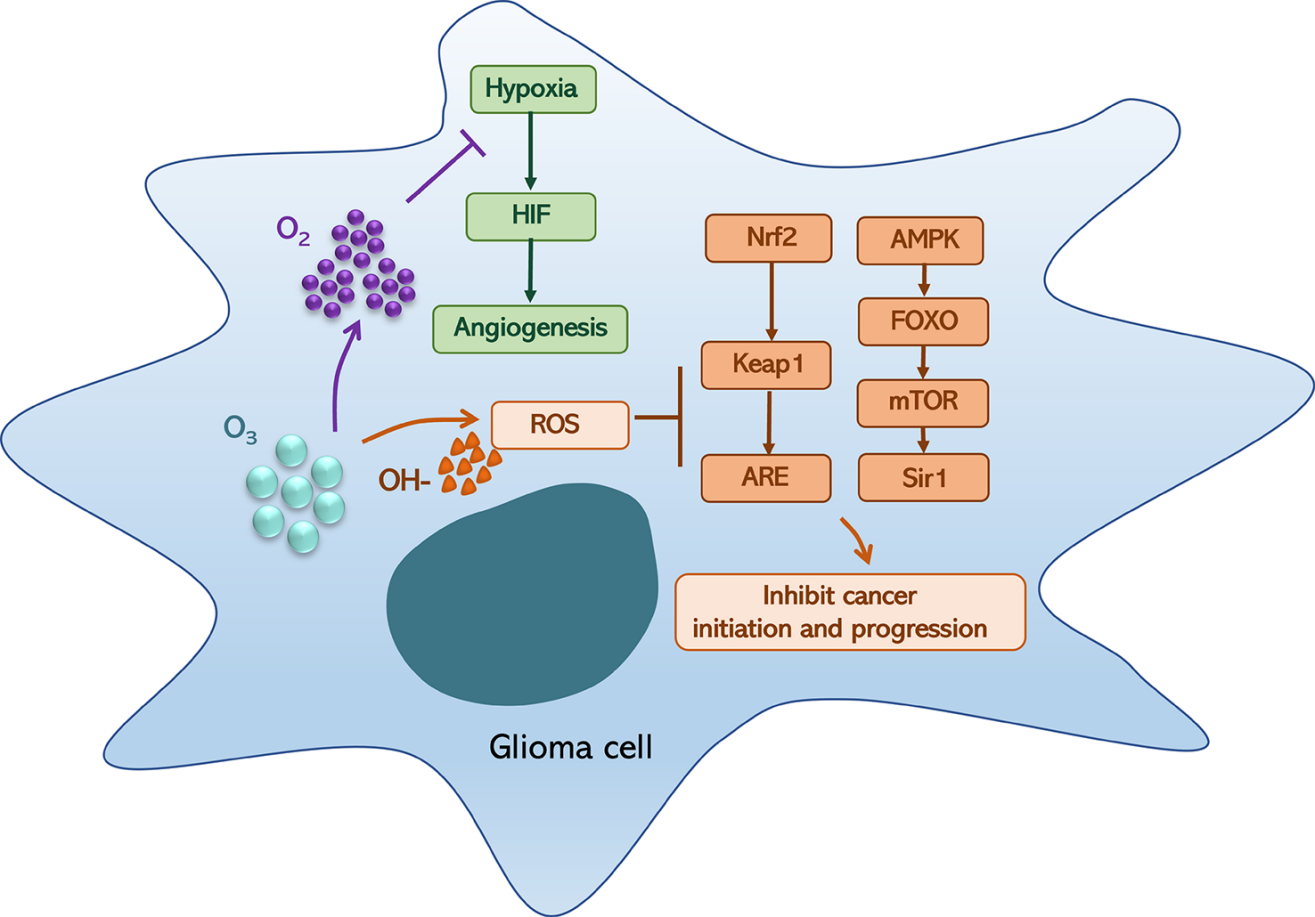
Ozone (O_3_) can generate hydroxyl radical (OH-) and oxygen (O_2_), which could induce a *reactive oxygen species* (ROS) anticancer effect and relieve hypoxia. On the one hand, oxygen relieves hypoxia that could inhibit hypoxia-inducible factor (HIF) generation and angiogenesis; on the other hand, an OH-associated high-level ROS can block the activation of the Nrf2/Keap1/ARE and AMPK/FOXO/mTOR/Sir1 pathways, which could inhibit cancer initiation.

On the other hand, preclinical findings show that in lung, breast, and colon cancers, ozone therapy can not only be used as a potential new chemotherapy (24, 25) but also utilized in combination with chemotherapy to enhance its efficacy (6, 26–28). By increasing the permeability of the tumor microenvironment, ozone therapy may allow chemotherapeutic drugs to penetrate deeper into tumor tissue, which has been shown to be more effective in reducing the growth of cancer cells than chemotherapy alone. Interestingly, the results of these studies indicate that a low rather than high ozone dose may be regarded as a potential modifier for chemotherapy. Moreover, *in vitro* studies have shown that the anti-tumoricidal ability of ozone-combined X-rays (9, 29) is improved compared with radiation treatment (RT) alone, with ozone showing a radio-sensitizing effect when used with radiation, enhancing the radiosensitivity of transformed radioresistant tumor cells (30) (**Figure 1**).

## Safety and risks of ozone therapy

Despite the efficacy of ozone therapy, its potential adverse effects must be considered carefully. At the right dose, ozone seems to induce non-toxic effects in cells of normal tissues because the antioxidant system of the cells can typically handle mild ozone-induced injury. In contrast, cancer cells have an almost exhausted antioxidant capacity (1). Ozone can be toxic at high concentrations and may cause serious side effects, such as pain, allergic reactions, hemolysis, and transient oxidative stress (31). Thus, it is important to evaluate potential benefits and risks before administering treatment.

Previous data have shown that, for ozone therapy, pain at the site of injection or insufflation is the most common side effect and usually resolves within a few hours. Allergic reactions are rare but can be serious; therefore, patients with a history of allergies should exercise caution when considering ozone therapy. Although rare, hemolysis is a potential concern; thus, patients with a history of anemia should be closely monitored during ozone therapy. Transient oxidative stress, while not harmful, is another potential side effect of ozone therapy; however, further research is required to determine the long-term effects of increased levels of oxidative stress (32). In other cases, ozone therapy may cause serious complications, such as infections, skin irritation, and lung damage (33, 34).

Meanwhile, an acute ozone exposure primarily affects glial cells in the central nervous system. In fact, ozone-induced vascular endothelial growth factor overexpression was observed in the astroglial cells of rat brainstems (35). In addition, damaged astrocytes were observed rapidly (2 and 4 h) after a dose of 60 mg/ml ozone was provided *in vitro (* 36), indicating a dose-dependent characteristic of the therapy.

## Preclinical data of ozone therapy in glioma treatment

Ozone therapy has been widely studied as a cancer treatment and affects the development of malignancy and poor prognosis. However, only a few preclinical studies have focused on ozone therapy for gliomas *in vitro* and *in vivo*. Basic research studies are summarized in **Table 1**.

**Table 1:** Preclinical studies utilizing ozone in treating glioma.

Author (year)	Study type	O_3_ application	Result	*Ref.
Zanker KS. et al. 1990	Basic research *In vitro*	Topical gas +/−5-FU	Glioma cell was not inhibited by O_3_, 5-FU, or both	(26)
Arizbeth Perez et al. 2014	Basic research *In vitro* and *in vivo*	*G1: ozone was dosed only once. G2: ozone was administered daily. G3: ozone was administered every 2nd day.	O3 is effective. Low ozone dosage was more effective than the higher dosage	(37)

*G, group; Ref, reference.

In 1990, Kurt S. Zänker and Ronald Kroczek found that ozone had a similar effect as chemotherapeutic drugs in increasing ROS inside cultured cells. The authors were the first to report ozone treatment for gliomas (26). Three fresh human glioma cells were cultured and incubated for 24 h with 5-FU and ozone in different concentrations (5-FU: 26, 50, and 100 μg/mL, respectively; ozone: 0.1, 0.2, 0.5, and 1.0 ppm, respectively); however, results were insignificant, indicating that individual and combined ozone and 5-FU use had no effect on glioma cells (26). However, based on the glioma treatment guideline, 5-FU is unsuitable for the initial treatment of glioma; thus, these results may not be accurate and further studies are needed. In 2014, a study by Perez et al. reported novel results on ozone-treated gliomas that indicated that ozone had antitumor activity, including against gliomas, both *in vitro* and *in vivo* (37).

Rat C6 glioma cells were cultured in ozone medium. Ozonated culture medium (ozone: CM=1:1, CM: culture medium) was added at the beginning of the experiment, daily, and every second day in groups 1, 2, and 3, respectively. Cell culture proliferation was assessed by measuring DNA concentration, followed by performing a CyQUANT^®^ assay. Interestingly, results showed that ozone had ambivalent effects. When ozone was applied daily (group 2), ozone induced an increase in cell proliferation, and increases in DNA concentration and DB index were observed compared with those of the control group. This can be explained by ozone-based enhancement of cell proliferation. In contrast, ozone inhibited C6 cell proliferation when a single dose was administered, indicating a decrease in DNA concentration (37). Thus, this study indicated that a low ozone dose most effectively reduced the C6 cell concentration *in vitro*.

An *in vivo* C6 glioma xenograft model was established using athymic male mice. Ozone was administered every second day in group 1 and every fifth day in group 2. The results showed a more evident tissue necrotic effect in group 2, revealing that the slope of the tumor volume growth was lower than that in group 1, which exhibited a high degree of necrosis due to a high degree of ROS accumulation. Thus, ozone has a dose-dependent effect in the prevention of tumor volume growth and inhibition of accelerated initial tumor growth. Furthermore, when investigating the therapeutic mechanism of ozone, this study found that concentrations of cholesterol and triglycerides were associated with effects of ozone. Although cholesterol and triglyceride concentrations were reduced by 40% and 25%, respectively, at high ozone dosages, the effect increased to 50% and 30%, respectively, for low ozone doses. Thus, low rather than high ozone dosage (*in vivo*) may be most effective for treating tumors *via* the regulation of ROS concentration (37). Therefore, for ozone dosages, higher is not necessarily better, and an equilibrated ozone dose may be more effective in inhibiting tumor activity than higher ozone doses.

## Clinical evidence of ozone therapy in the treatment of high-grade glioma

According to promising preclinical data, ozone should be effective in treating gliomas, especially when equilibrated ozone doses are used. However, the use of ozone therapy for glioma treatment remains controversial. The limited number of clinical studies and contradictory results from these studies highlight the need for further research to fully evaluate the potential benefits and limitations of ozone therapy for glioma (38).

In 2018, Clav et al. reported inconclusive findings of a clinical trial (6, 22). Seven patients with high-grade glioma were enrolled after surgery or tumor biopsy between 2005 and 2008. All patients received ozone treatment *via* autohemotherapy combined with a concurrent STUPP protocol. This protocol, consisting of TMZ combined with radiotherapy treatment for GBM, was developed by Roger Stupp in 2005 and has become the standard of care for high-grade gliomas (39). According to a clinical trial that enrolled six patients with GBM (grade IV), no difference in overall survival (OS) was observed between those treated *via* STUPP with and without ozone. In patients with anaplastic astrocytoma (AA, grade III) who underwent STUPP with ozone, one patient survived 11 years with a good quality of life (Karnofsky, KPS 100%). Unfortunately, the study was terminated early because of difficulties with recruitment (6, 22).

Another 2018 study by Megele et al. reported five cases of recurrent GBM between 2012 and 2013 (38). Patients diagnosed with GBM were offered intratumoral O_2_–O_3_ treatment concurrent with the STUPP protocol. While a median of 27 (range, 3–44) O_2_–O_3_ applications were administered to the patients, the median overall survival (mOS) rate was 40 months (range, 16–53 months) and the mOS following the first recurrence subsequent to the initiation of O_2_–O_3_ treatment was 34 months (range, 12–53 months). Furthermore, one patient survived more than 4 years after diagnosis. Clinical studies of ozone treatment for gliomas are shown in **Table 2**.

**Table 2:** Clinical studies utilizing ozone in treating glioma.

Author (year)	Year	Pat. No.	Treatment	Outcome	*Ref.
Bernardino Clav et al.	2018	*6 GBM (G: IV) *1 AA* (G: III)	STUPP + ozone	GBM: negative AA: positive	(21)
Richard Megele et al.	2018	*5 recurrent GBM (G: IV)	1^st^ L: STUPP + ozone 2^st^ L: PCV + ozone	Positive	(38)

*GBM, glioblastoma; AA, anaplastic astrocytoma; G, grade; Ref, reference.

Despite the conflicting results reported in aforementioned studies (22, 38), there is growing interest in the use of ozone therapy for the treatment of gliomas, and research is being conducted to further explore its potential benefits and limitations.

## Discussion

The STUPP strategy for treating gliomas, particularly high-grade gliomas, involves a maximally safe surgical resection, followed by concurrent RT and TMZ for 6 weeks and adjuvant TMZ for 6 months thereafter. Previous clinical trials assessing the treatment of newly diagnosed patients with GBM concluded that RT plus TMZ compared with RT alone significantly improved overall survival (OS) (14.6 vs. 12.1 months) and progression-free survival (PFS) at 6 months (53.9% vs. 36.4%). Moreover, lomustine, intravenous carmustine, carmustine wafer implants, bevacizumab, and tumor treatment fields (TTFields) have been approved by the FDA and are used to treat high-grade gliomas. TTFields, in particular, is the only treatment that showed improvement based on OS (20.5 vs. 15.6 months) and PFS6 (56% vs. 37%) compared to the current standard treatment (40–42). Meanwhile, for recurrent GBM, re-radiotherapy is the main strategy. A clinical study by Kazmi et al. showed that administering re-radiotherapy for recurrent GBM resulted in OS-6 and OS-12 rates of 73% (95% CI 69%–77%) and 36% (95% CI 32%–40%), respectively; PFS-6 and PFS-12 rates of 43% (95% CI 35%–50%) and 17% (95% CI 13%–20%), respectively; and a Grade 3^+^ adverse event (AE) rate of 7% (95% CI 4%–10%) (43). Overall, it is apparent that the standard treatment for high-grade glioma yields unsatisfactory results (39), which has led to the emergence of ozone therapy as a promising treatment for cancer. In this study, we discussed the application of ozone treatment for gliomas.

Based on our findings, photon radiation therapy combined concurrently with ozone treatment can regulate PD-1 activation in the tumor microenvironment and enhance the effects of radiation by promoting OH generation in breast cancer (5). Moreover, glioma tissue is in a hypoxic environment, with aerobic glycolysis the most significant sign of tumor formation (Warburg effect); thus, ozone may improve oxygen conditions and enhance radiation treatment of glioma. Therefore, we propose that enhancement of radiation therapy for glioma should be more pronounced than that of other tumors, making the therapy suitable for treatment of gliomas.

Recent preclinical and clinical trials suggest that ozone therapy is potentially a valuable adjuvant regimen for the treatment of gliomas. However, only a few clinical trials have focused on the use of ozone for glioma treatment. Among them, contradictory outcomes have been reported. A clinical trial by Clav et al. produced negative results, whereas a study by Megele et al. showed positive findings. Megele et al. reported that ozone therapy involves the injection of an ozone/oxygen mixture into the tumor concurrent with a standard glioma treatment protocol (38). In the study, the mOS from diagnosis to the first recurrence and from the first recurrence to the initiation of the O_2_–O_3_ treatment was 40 and 34 months, respectively. In that study, one patient survived more than 4 years after diagnosis, demonstrating the effectiveness of ozone in treating glioma; however, only non-randomized controlled trials (RCTs) have been performed to investigate the effectiveness of ozone treatment with concurrent radiation and/or chemotherapy. To date, no RCTs have been conducted, and insufficient evidence exists regarding the systematic use of ozone in anticancer treatment (6).

In addition to previous studies, further preclinical studies should evaluate effects of the drug delivery method, whereas further clinical trials should investigate the effects of timing and dosage of ozone administration concurrent with the STUPP protocol.

## Conclusion

In conclusion, ozone therapy represents a promising alternative treatment option for those with cancer and may be a viable regimen for use against gliomas. However, the exact mechanism of action of ozone therapy in gliomas is not yet understood and there is limited evidence in literature. Further basic studies and clinical trials are required to fully evaluate the potential benefits and limitations of ozone therapy.

## Author contributions

LY and WF contributed to the conception of the review and participated in data collection and drafting of the manuscript. PR and CR supervised data collection and drafted and revised the manuscript. ZL assisted with conducting the study and collected the data. All authors contributed to the article and approved the submitted version.

## References

[1] Brinkman HBL R. Ozone as a possible radiomimetic gas. Nature (1958) 181:1202–3. doi: 10.1038/1811202a0 13541419

[2] HH W. Die behandlung peripherer durchbutungsstorungen mit ozon. Erfahr Hk (1974) 23:181–4.

[3] Masaru Sagai VB. Mechanisms of action involved in ozone therapy: is healing induced *via* a mild oxidative stress? Med Gas Res (2011) 1:29. doi: 10.1186/2045-9912-1-29 22185664 PMC3298518

[4] Fetner RH. Ozone-induced chromosome breakage in human cell cultures. Nature (1962) 194:793–4. doi: 10.1038/194793a0 13892647

[5] Dan Zheng YL Song L Xu T Jiang X Yin X He Y. Improvement of radiotherapy with an ozone-carried liposome nano-system for synergizing cancer immune checkpoint blockade. Nano Today (2022) 47:101675. doi: 10.1016/j.nantod.2022.101675

[6] Clavo B Santana-Rodriguez N Llontop P Gutiérrez D Suárez G López L. Ozone therapy as adjuvant for cancer treatment: is further research warranted? Evid Based Complement Alternat Med (2018) 2018:7931849. doi: 10.1155/2018/7931849 30271455 PMC6151231

[7] Sweet M-SK F Lee S-CD Hagar WL Sweet WE. Ozone selectively inhibits growth of human cancer cells. Science (1980) 209:931–3. doi: 10.1126/science.7403859 7403859

[8] Agarwal DB. Role of ozone therapy and cancer: myth or reality? J Med Sci And Clin Res (2019) 7(7):720–27. doi: 10.18535/jmscr/v7i7.127

[9] Fetner RH. Chromosome breakage in vicia faba by ozone. Nature (1958) 181:504–5. doi: 10.1038/181504a0

[10] Lerner RA Eschenmoser A. Ozone in biology. PNAS (2017) 100(6):3013–15. doi: 10.1073/pnas.0730791100' PMC152232 12631693

[11] David C.Bolton BKT ChungZee Y Osebold JW. An *in vitro* system for studying the effects of ozone on mammalian cell cultures and viruses. Environ Res (1982) 27(2):466–75. doi: 10.1016/0013-9351(82)90101-3 7084169

[12] Scassellati C Galoforo AC Bonvicini C Esposito C Ricevuti G. Ozone: a natural bioactive molecule with antioxidant property as potential new strategy in aging and in neurodegenerative disorders. Ageing Res Rev (2020) 63:101138. doi: 10.1016/j.arr.2020.101138 32810649 PMC7428719

[13] Théophile A Mobio RA Baudrimont I Carratú M-R Shier TW Dano SébastienD. Epigenetic properties of fumonisin B1: cell cycle arrest and DNA base modification in C6 glioma cells. Toxicol Appl Pharmacol (2000) 164(1):91–6. doi: 10.1006/taap.2000.8893 10739748

[14] Yesennia Sánchez-Pérez CC-L García-Cuellar C Pérez-Carreón J Hernández-García S Salcido-Neyoy M Alemán-Lazarini L. Oxidative stress in carcinogenesis. correlation between lipid peroxidation and induction of preneoplastic lesions in rat hepatocarcinogenesis. Cancer Letter (2005) 217(1):25–32. doi: 10.1016/j.canlet.2004.07.019 15596293

[15] Salazar-Ramiro A Ramirez-Ortega D Perez de la Cruz V Hérnandez-Pedro NY González-Esquivel DF Sotelo J. Role of redox status in development of glioblastoma. Front Immunol (2016) 7:156. doi: 10.3389/fimmu.2016.00156 27199982 PMC4844613

[16] Sanchez-Perez Y Soto-Reyes E Garcia-Cuellar CM Cacho-Diaz B Santamaria A Rangel-Lopez E. Role of epigenetics and oxidative stress in gliomagenesis. CNS Neurol Disord Drug Targets (2017) 16(10):1090–8. doi: 10.2174/1871527317666180110124645 29318979

[17] Gill JG Piskounova E Morrison SJ. Cancer, oxidative stress, and metastasis. Cold Spring Harb Symp Quant Biol (2016) 81:163–75. doi: 10.1101/sqb.2016.81.030791 28082378

[18] Reuter SCG S Chaturvedi MM Aggarwal. Oxidative stress BB. Inflammation, and cancer: how are they linked? Free Radical Biol Med (2010) 49(11):1603–16. doi: 10.1016/j.freeradbiomed.2010.09.006 PMC2990475 20840865

[19] Cenci A Macchia I La Sorsa V Sbarigia C Di Donna V Pietraforte D. Mechanisms of action of ozone therapy in emerging viral diseases: immunomodulatory effects and therapeutic advantages with reference to SARS-CoV-2. Front Microbiol (2022) 13:871645. doi: 10.3389/fmicb.2022.871645 35531273 PMC9069003

[20] Hayes JD Dinkova-Kostova AT Tew KD. Oxidative stress in cancer. Cancer Cell (2020) 38(2):167–97. doi: 10.1016/j.ccell.2020.06.001 PMC7439808 32649885

[21] Clavo B Ruiz A Lloret M López L Suárez G Macías D. Adjuvant ozonetherapy in advanced head and neck tumors: a comparative study. Evid Based Complement Alternat Med (2004) 1(3):321–5. doi: 10.1093/ecam/neh038 PMC538509 15841266

[22] Bernardino Clavo JLP López L Suárez G Lloret M Rodríguez V Macías D. Ozone therapy for tumor oxygenation: a pilot study. Evidence-Based Complementary Altern Med (2004) 1(1):93–8. doi: 10.1093/ecam/neh009 PMC442111 15257330

[23] Schulz S Haussler U Mandic R Heverhagen JT Neubauer A Dünne AA. Treatment with ozone/oxygen-pneumoperitoneum results in complete remission of rabbit squamous cell carcinomas. Int J Cancer (2008) 122(10):2360–7. doi: 10.1002/ijc.23382 18224691

[24] Baeza-Noci J Pinto-Bonilla R. Systemic review: ozone: a potential new chemotherapy. Int J Mol Sci (2021) 22(21):1–6. doi: 10.3390/ijms222111796 PMC8584016 34769225

[25] Simonetti VQ V Giustetto P Franzini M Iaffaioli RV. Association of ozone with 5-fluorouracil and cisplatin in regulation of human colon cancer cell viability: *In vitro* anti-inflammatory properties of ozone in colon cancer cells exposed to lipopolysaccharides. Evidence-Based Complementary Altern Med (2017) 2017:1–6. doi: 10.1155/2017/7414083 PMC5868048 29721026

[26] Kurt S Zänker RK. *In vitro* synergistic activity of 5-fluorouracil with low-dose ozone against a chemoresistant tumor cell line and fresh human tumor cells. Chemotherapy (1990) 36(2):147–54. doi: 10.1159/000238761 2311442

[27] Cannizzaro CVF A Martinelli M Misiti S Brunetti E Bucci B. O2/3 exposure inhibits cell progression affecting cyclin B1/cdk1 activity in SK-N-SH while induces apoptosis in SK-N-DZ neuroblastoma cells. J Cell Physiol (2007) 213(1):115–25. doi: 10.1002/jcp.21097 17477375

[28] Luongo M BA Mascolo L Gaudino G. Possible therapeutic effects of ozone mixture on hypoxia in tumor development. Anticancer Res (2017) 37:425–35. doi: 10.21873/anticanres.11334 28179287

[29] Fetner RH. Ozone-induced chromosome breakage in human cell cultures. Nature (1962) 194:793–4. doi: 10.1038/194793a0 13892647

[30] Karlic H Kucera H Metka M Schönbauer M Söregi G. Effect of ozone and ionizing radiation on an *in vitro* model–a pilot study of 4 gynecologic tumors. Strahlenther Onkol (1987) 163):37–42. 3810475

[31] Elvis AM Ekta JS. Ozone therapy: a clinical review. J Nat Sci Biol Med (2011) 2(1):66–70. doi: 10.4103/0976-9668.8231 PMC3312702 22470237

[32] Baeza-Noci J Pinto-Bonilla R. Systemic review: Ozone: A potential new chemotherapy. Int. J. Mol. Sci. (2021) 22(21):11796. doi: 10.3390/ijms222111796 34769225 PMC8584016

[33] Mudway FJK IS. Ozone and the lung: a sensitive issue. Mol Aspects Med (2000) 21(1):1–48. doi: 10.1016/s0098-2997(00)00003-0 10804262

[34] Valacchi VF G Bocci V. The dual action of ozone on the skin. Br J Dermatol (2005) 153(6):1096–100. doi: 10.1111/j.1365-2133.2005.06939.x 16307642

[35] Araneda S Commin L Atlagich M Kitahama K Parraguez VH Pequignot JM. VEGF overexpression in the astroglial cells of rat brainstem following ozone exposure. Neurotoxicology (2008) 29(6):920–7. doi: 10.1016/j.neuro.2008.09.006 18848842

[36] Z-jF N-bZ Sun T. Effects of different concentrations of oxygen – ozone on rats’ astrocytes *in vitro* . Neurosci Lett (2008) 441(2):178–82. doi: 10.1016/j.neulet.2008.06.036 18577417

[37] Perez A Santos Cuevas CL Chairez I Poznyak T Ordaz-Rosado D García-Becerra R. Ozone dosage effect on C6 cell growth: in vitro and in vivo tests. Anticancer Agents Med Chem (2015) 15(9):1190–6. doi: 10.2174/1871520614666141027143914 25353336

[38] Megele R Riemenschneider MJ Dodoo-Schittko F Feyrer M Kleindienst A. Intra-tumoral treatment with oxygen-ozone in glioblastoma: a systematic literature search and results of a case series. Oncol Lett (2018) 16(5):5813–22. doi: 10.3892/ol.2018.9397 PMC6176341 30344733

[39] Catarina Fernandes AC Osório Lígia Lago RC Linhares P Carvalho B Caeiro Cláudia. Current standards of care in glioblastoma therapy. In: S DV, editor. Glioblastoma. Brisbane (AU): Codon Publications (2017). 29251860

[40] Fisher JP Adamson DC. Current FDA-approved therapies for high-grade malignant gliomas. Biomedicines (2021) 9(3):1–13. doi: 10.3390/biomedicines9030324 PMC8004675 33810154

[41] Annika Malmström BHG Marosi C Stupp R Frappaz D Schultz H Abacioglu U. Nordic Clinical brain tumour study group. temozolomide versus standard 6-week radiotherapy versus hypofractionated radiotherapy in patients older than 60 years with glioblastoma: the Nordic randomised, phase 3 trial. Lancet Oncol (2012) 13(9):916–26. doi: 10.1016/S1470-2045(12)70265-6 22877848

[42] Blumenthal DT Gorlia T Gilbert MR Kim MM Burt Nabors L Mason WP3. Is more better? the impact of extended adjuvant temozolomide in newly diagnosed glioblastoma: a secondary analysis of EORTC and NRG Oncology/RTOG. Neuro Oncol (2017) 19(8):1119–26. doi: 10.1093/neuonc/nox025 PMC5570239 28371907

[43] Kazmi F Soon YY Leong YH Koh WY Vellayappan B. Re-irradiation for recurrent glioblastoma (GBM): a systematic review and meta-analysis. J Neurooncol (2019) 142(1):79–90. doi: 10.1007/s11060-018-03064-0 30523605

